# A Few Thoughts on Epilepsy

**Published:** 1857-04

**Authors:** 


### ECLECTIC DEPARTMENT,

### AND SPIRIT OF THE MEDICAL PERIODICAL PRESS.

---

*A Few Thoughts on Epilepsy.* By L. M. Lawson, M. D., Professor of the Theory and Practice of Medicine, in the Medical College of Ohio.

This disease (which has well nigh been ranked among the *opprobria medicinæ,*) has of late years received a large share of attention from practitioners and pathologists. But notwithstanding these laborious and extensive investigations, it still remains (so to speak) a *terra incognita.* It is to us, as it was to our predecessors, a *morbus herculeus* or a *morbus sacer*—too often defying our most cherished remedies, mocking alike the skill of the physician and the miseries of the patient.

But in the midst of this darkness and confusion, a few glimmering rays of light now and then steal in upon us; and while, in some respects, they serve only to heighten the horrors of the scene, they do reveal a few tangible points, which may assist us in reaching more rational conclusions.

We cannot say that epilepsy has, in fact, any *pathological anatomy;* indeed, the morbid changes which have been observed after death, exhibit but little uniformity, and therefore cannot be regarded as essential to the disease. Probably the only condition which throws much light on the malady, is the state of *atrophy* observed in the cortical portion of the brain in old epileptics. This condition indicates a derangement of the *nutrition* of the part, the result of *diminished* action.

The low state of therapeutics in relation to epilepsy, may be traced to two causes:

1st. The absence of any recognizable pathological anatomy; and,

2d. An effort to supply this great hiatus by the use of *specifics.* Hence, even our French confrères, who usually exhibit so much philosophy and careful discrimination, do little more than employ, in the most routine manner, some fancied specific. Trousseau, for example, finally fixes on the belladonna treatment, which he thinks must be continued from two to four years.

Dr. Todd, of London, has recently attempted to bridge over this great chasm, by suggesting that a *polarized* state of the brain (or certain parts of it) occur, and that it is the "disruptive discharge" (chemically speaking) which induces the paroxysm. He recognizes two degrees; one involving the cerebral hemispheres, leading to loss of consciousness and impaired intellect; the other, more profound, extending to the tubercula quadrigemina and mesocephate, causing the convulsions. All this he ascribes to a change of *nutrition* of the part, which induces a highly charged or polarized state, and the tension which ensues leads ultimately to the *disruptive discharge*, and all the phenomena of the paroxysm. He does not admit that inflammation, congestion, or anæmia produce the disease; but that it often arises from the specific influence of some poison circulating in the blood, among which may be mentioned the influence of retarded urea.

These experiments, however, are only feeble approaches toward an explanation; for, although they convey some faint idea of what may possibly be occurring, they are so dim and shadow like as to leave the inquirer still faltering and undecided. The only suggestion of *practical* value, embraces the idea that the foundation of the disease is essentially deranged *nutrition*.

Dr. Radcliffe has offered an explanation of the principal phenomena which occur in epilepsy and analogous diseases, which embraces as a fundamental idea *diminished* nervous action. According to this view, the spasmodic action is due to diminished circulation and innervation, in consequence of which molecular contraction of the muscles ensues. Spasmodic action, therefore, is due to the *withdrawal* of stimulants, instead (as was before believed) an increase of excitement.

The *novelty* of this doctrine will secure for it a hearty rejection by those who are accustomed to think only in a single channel; nevertheless, there are various facts and analogies (which cannot now be enumerated) which strongly favor such a conclusion, if they do not positively establish it. Let me name but one—by no means the most conclusive: We can *bleed* a patient into, first, syncope; second, *convulsions*. Here is diminished excitement followed by violent muscular contraction. Is that contraction due to an increase or diminution of excitement? But one answer can be returned.

Independent of either of these theories, I am led to believe that the essential pathology of epilepsy is *diminished* action, probably connected (at least ultimately) with impaired nutrition. It may, however, arise from the action of poisons, from sympathetic influence, or direct derangement. It is, in fact, connected with impaired vitality generally, such as sluggish innervation, circulation, secretions, mental manifestations, and so on. All this may result from onanism, retained urea, the elements of bile or other poisons, sympathetic derangement of the alimentary canal and uterus, and excessive and exhausting mental efforts or emotions. I each instance, however, the *ultimate* effect (and that which leads to regularly developed epilepsy) is exhausting in its character, and finally impairs nutrition.

It is a peculiar fact, and one which is very significant in this connection, that an intercurrent excitement—such as fever or inflammation—temporarily suspends epilepsy, which could hardly happen if the disease was not one of diminished vitality. An instance has come to my knowledge in which a severe fall, from some considerable height, had the same effect.

The conclusion which appears to me most in accordance with the phenomena of epilepsy is, that the essential, pathological state is one of depressed vitality, including impaired nutrition. But while this is admitted, we must often look beyond the nervous system for some exciting or predisposing cause; and hence there is some justness in the following varieties of epilepsy, as enumerated by Dr. Cheyne; epilepsia *cerebralis*, *sympathetica stomachica*, *hepatica*, *nervosa*, *uterina*, *à dolore*. It must be remembered, however, that these functional derangements may be a consequence and not the cause of an attack.

In regard to the urinary secretion, it has been shown by Dr. Hunt and Dr. Todd, that certain derangements of this function may lead to epilepsy, and that its cure consists in correcting that condition; and the same remarks apply with equal force to the hepatic function. Thus, Dr. Todd found the urine albuminous, with deficient urea; Dr. Hunt observed the secretion to manifest a feeble acid reäction, with low specific gravity, increase of mineral ingredients with diminution of urea and other organic matter. These facts, coupled with the observations of Dr. Prout, that when soda and ammonia are in excess, urea becomes diminished, and that soda and potash seriously injure the nervous system; and we have a key to at least some of the morbid changes which occur in such cases.

But, after all, the great object is to find a successful mode of *treatment.* Unfortunately, most of the *specifics* have failed, or, if occasionally successful, they are not so with sufficient uniformity to establish for either one a particular preëminence. Thus, nitrate of silver, oxyde of zinc, sulphate of copper, iron, arsenic, digitalis, valerianate of zinc, belladonna, the recently vaunted cotyledon umbilicus—and an innumerable host of similar agents—have each in their turn signally failed. It is true, each may at times succeed, but their application is altogether empirical, and therefore necessarily unsuccessful.

In view of the depressed state of vitality, the leading indication with me is to improve the tone of the system by the administration of stimulants, tonics and suitable nourishment. For this purpose I employ brandy, iron and animal food. These agents improve the tone of the system, and I have witnessed the suspension (and probable cure) of the disease while under this course of treatment. In one case of six years' duration, violent paroxysms occurring at least weekly, with the "*petit mal*" almost daily, the paroxysms were subdued, and the patient apparently restored, under the use of ounce doses of brandy three times a day, together with purgatives, and small doses of hyoscyamus and strychnine.

But I would by no means neglect the kidneys and liver. If there is diminished urea, employ diuretics; if the alkalies have increased in the urine, give acids, especially the nitro-muriatic. If the liver is torpid (which is almost invariably the case), give cathartics, containing more or less mercury. I place great reliance on the judicious (but decided) employment of cathartics; but none of these agents should interfere with the tonic and stimulating treatment. Restore the general health by means of alteratives and tonics, and by keeping the renal and hepatic emunctories acting freely, and then overcome the nervous disease by cardiac and nervous stimulants.

Among the class of remedies known as *specifics* (so called because their mode of action is unknown), none has gained more reputation than the *oxide of zinc.* M. Herpin (who received a prize from the Institute of France, in 1850, for his treatise on this subject) eulogises the virtues of the zinc, and indeed placed his chief reliance on that agent. He gave the zinc in doses of six to eight grains daily, in divided portions, augmented every week by two grains daily, until the dose reached *forty-five grains!* This was continued for months in succession. The nausea, which appeared at first, soon passed off, and no further inconvenience from the large doses was experienced. He mentions one remarkable case in which 1200 fits had occurred, cured by a combination of zinc and belladonna. Of 40 cases treated with the oxyde of zinc alone, 28 were cured—a far greater success than has fallen to the lot of most practitioners.

It does not appear, however, that others have been equally successful with this agent. Thus, M. Trousseau, in the very face of Herpin's experiments, abandons the zinc, and now relies mainly on belladonna. He employs a pill composed of the powder and extract of the roots of belladonna *aa* one-seventh grain. One pill is given every night for the first month, which should be increased one for each month up to the fourth month. He has cured 20 out of 150 cases; and M. Blache has had about similar success.

In regard to prognosis, it may be remarked that the principal elements are age, duration of the disease, frequency of paroxysm, &c. The periods most susceptible of cure, are, first, between 10 and 20 years, and, second, above fifty; from 20 to 30 being the most unfavorable. It is an important fact mentioned by Herpin, that when the paroxysms have not exceeded 100, he cured 74 per cent.; but when they exceeded 500, he did not succeed in curing a case.

One of the most important elements of success in treatment, is *perseverance.* Trousseau remarks that if, at the end of one year, a little mitigation can be perceived, there is ground for encouragement; but that the medicine must usually be continued from two to four years. I am fully persuaded that our want of success often depends on the frequent change of remedies, and the two early abandonment of even a correct course. We should study well our case, become perfectly sure of its essential points, and then adopt such course as promises most success, *and persevere to the end.* No doubt it was in this way that M. Herpin secured a higher per centage of cures than his cotemporaries, and it is by the same unfaltering course that others can obtain a like success.

Finally, I would enumerate the following points in the treatment:

1. Cathartics, more or less mercurial.
2. Tonics, especially iron.
3. Acids or diuretics, in deranged urinary secretion.
4. Stimulants, brandy, whisky, gin.
5. The special agents, oxyde of zinc being preferable.
6. Belladonna, combined with the oxyde of valerianate of zinc, in special cases.
7. Above all, *persevere to the end.*— *Western Lancet.*

